# Relationship between thigh muscle cross-sectional areas and single leg stand-up test in Japanese older women

**DOI:** 10.1371/journal.pone.0269103

**Published:** 2022-06-14

**Authors:** Keiko Kishigami, Hiroaki Kanehisa, Shumeng Qi, Takuma Arimitsu, Motohiko Miyachi, Motoyuki Iemitsu, Kiyoshi Sanada

**Affiliations:** 1 College of Sport and Health Science, Ritsumeikan University, Shiga, Japan; 2 National Institute of Fitness and Sports in KANOYA, Kagoshima, Japan; 3 Faculty of Health Care, Department of Human Health Hachinohe Gakuin University, Aomori, Japan; 4 Faculty of Sport Sciences, School of Sport and Sciences, Waseda University, Tokyo, Japan; 5 Department of Health Promotion and Exercise, National Institute of Health and Nutrition, Tokyo, Japan; University of Mississippi, UNITED STATES

## Abstract

In older adults, the quantitative decline of the quadriceps femoris is associated with the augmentation of difficulty in the execution of a stand-up task. However, it is unclear whether the cross-sectional areas (CSAs) of individual thigh muscles differ between older adults who can stand up from a 40-cm-height chair on a single leg and those who cannot. To investigate this, the present study determined the CSAs of individual mid-thigh muscles in 67 Japanese women aged 60–77 years by using a magnetic resonance imaging method. Participants were asked to stand up from a 40-cm-height chair on a single leg, and those who could and could not stand up without leaning back and maintain a standing posture for 3 seconds on a single leg were allocated into the successful group (SG, n = 40) and unsuccessful group (USG, n = 27), respectively. Only the CSA of the adductors (sum of the adductor longus and adductor magnus) was significantly smaller in USG compared to SG. When CSA was expressed relative to the two-third power of body mass, the values for the four heads of the quadriceps femoris and biceps femoris long head, as well as the adductors, were significantly lower in USG than in SG. The current results indicate that in terms of the value relative to body mass, the reduced CSAs of the adductors and biceps femoris long head, as well as the four heads of the quadriceps femoris, are associated with the failure of attempts to stand up from a 40-cm-height chair on a single leg in older women. This may be due to the anatomical function of the two muscle groups, which contributes to hip extension movement involved in transitioning from a sitting position to a standing position during the stand-up task.

## Introduction

Standing up from a chair to an upright posture is one of the most demanding daily activities in mechanical terms [1]. The physical capacity required to perform a stand-up task without difficulty is essential for independent living [2]. The lowest height from which one can stand up is an independent parameter associated with fall risk in older adults [3]. Thus, a chair stand-up task is extensively adopted as a representative test to assess the ability of older adults to perform activities of daily living (ADL). Notably, a 40-cm-height chair stand-up test with one leg has drawn attention as a means for the early detection of locomotive syndrome [4], being physical conditions requiring nursing care services due to problems relating to locomotive organs or having risks which may lead to the need for the nursing care services in the future [5]. If one cannot stand up from a 40-cm-height chair on a single leg, the person is diagnosed with stage 1 locomotive syndrome [6]. To our knowledge, however, no study has examined how the size of individual thigh muscles influences the success or failure of the single-leg stand-up task.

For elderly individuals, the reduced force-generation capacity of quadriceps femoris is linked to the augmentation of efforts to perform ADL, including the movement to stand up from a chair in older adults [7–11]. In older women, however, sit-to-stand performance power, calculated from hip maximum velocity during standing up multiplied by body weight, is more closely associated with thigh muscle volume than isometric quadriceps strength [12]. This suggests the contribution of thigh muscles other than the quadriceps femoris to the execution of stand-up task. Stand-up movement consists of the following three phases: preparation, ascending, and stabilization [13]. The series of motions from the preparation phase to the ascending phase include forward leaning of the trunk, followed by seat-off events to move from a sitting position to a standing position [13]. Considering these aspects, it is assumed that the amount of thigh muscles other than the quadriceps femoris, which are involved in the hip extension, will also be a factor associating with the success or failure of the stand-up task without support.

EMG recordings have shown that the hamstrings as well as the quadriceps remain active throughout the lifting phase of the sit-to-stand motion [14, 15]. Furthermore, previous studies using magnetic resonance imaging (MRI) [16, 17] or EMG recording [18] provided evidence indicating that the adductors also contribute to the execution of movements, including hip extension. Based on these findings, it is reasonable to assume that the size of the hamstrings and adductors, as well as the quadriceps femoris, will influence the success/failure of the stand-up task without support in older adults. Examining this may provide useful information for designing exercise programs improving the ability of alder adults to perform the stand-up motion.

In general, the measured muscle strength and/or power vary with the procedures used to determine them, even if the muscle groups involved in the determinations are the same [19]. On the other hand, the force generation capacity of a muscle is related to its cross-sectional area (CSA) [20]. For middle-aged and elderly individuals, not only isometric strength but also the CSA of the quadriceps femoris has been shown to be significantly correlated to power index, being derived from body mass, acceleration of gravity, leg length, and time taken for a sit-to-stand test using a chair 40-cm-height [21]. In this case, the magnitude of correlation coefficient was similar between the two parameters of the quadriceps femoris [21]. By using CSA, therefore, the present study aimed to examine differences in the muscularity of individual mid-thigh muscles between older women who can stand up from a 40-cm-height chair on a single leg and those who cannot. We hypothesized that CSAs of the hamstrings and adductors, as well as the quadriceps femoris, would be smaller in women who cannot complete the stand-up task as compared to those who can.

## Methods

### Participants

This study was conducted as one program of a research project named “Health Survey of Old Women at Ritsumeikan University in Kusatsu City, Shiga Prefecture”. The participants were recruited from the residents of Kusatsu City, being one of the major cities in Shiga prefecture and locates about 20 km from Kyoto City. Kusatsu City has a population of 144,349 in 2021, in which the residents aged 65 and over is 30,750 (the proportion to the total residents: 21%). We recruited women aged 60 years and more as participants in this study. Sixty-seven Japanese women aged 60–77 years voluntarily participated in this study. Means ± standard deviations (SDs) of age, height, body weight, body mass index (BMI), and waist circumference were 67.6 ± 5.4 years, 155.7 ± 5.6 cm, 55.4 ± 8.7 kg, 22.8 ± 3.2 kg/m^2^, and 85.6 ± 9.8 cm, respectively. No one of the participants had a history of disease such as CNS disorders or musculoskeletal diseases affecting the execution of stand-up task from a chair with both legs. In addition, all were functionally independent in daily life and were not on extreme diets or major medications (e.g., chemotherapy, cardiac/respiratory/antipsychotic drugs). Participants were allocated to the successful group (SG) and unsuccessful group (USG) based on the success/failure to complete the single-leg stand-up task. The purpose, procedures, and risks of the study were explained to all participants before obtaining written informed consent. This study was approved by the Institutional Review Board of Ritsumeikan University (BKC-LSMH-2021-053).

### Muscle CSA measurements

**Fig 1:**
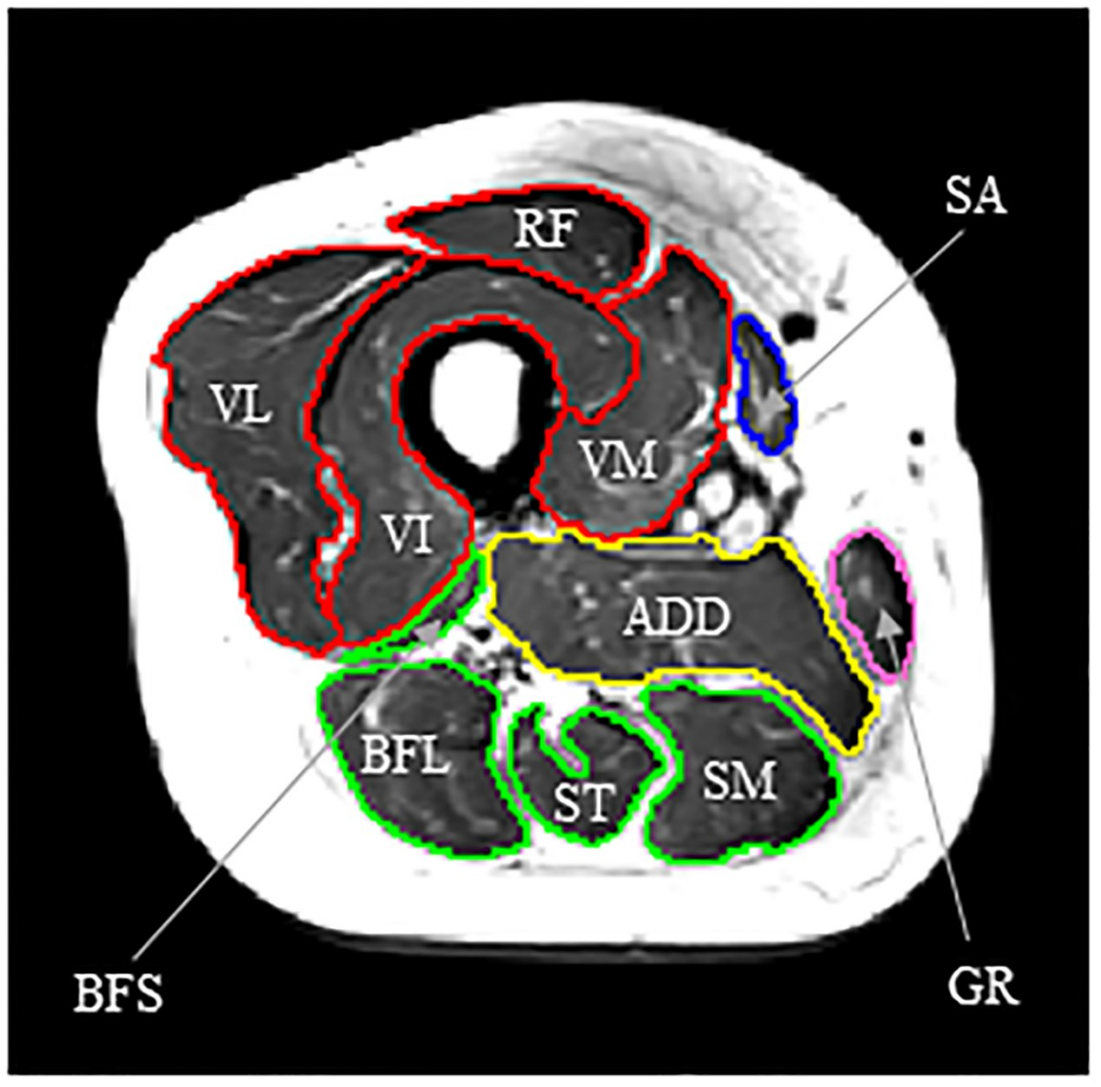
Cross-sectional image of the mid-thigh of a woman in USG (age, 67 years; height, 154.6 cm; body mass, 49.8 kg; BMI, 20.8 kg/m^2^). Areas surrounded by red, green, yellow, blue, and pink lines correspond to the respective thigh muscle groups, i.e., the quadriceps femoris (comprising the rectus femoris, RF; vastus lateralis, VL; vastus intermedius, VI; and vastus medialis; VM), hamstrings (comprising the biceps femoris short head, BFS; biceps femoris long head, BFL; semitendinosus, ST; and semimembranosus, SM), adductors (ADD), sartorius (SA), and gracilis (GR), respectively. Non-muscular elements (fat tissue within the muscle compartment, aponeurosis, blood vessels, nerves, and femur bone) were excluded to the extent possible in the manual segmentation.

After the completion of anthropometric measurements (height, body mass, and waist circumference), the cross-sectional images of the right and left thighs were obtained with a 3.0T MRI scanner (Magnetom Skyra, Siemens Healthineers, Erlangen, Germany). Imaging was performed from the trochanter minor down to the transition of the muscle rectus femoris to its tendon with an axial T1-weighted fast spin-echo technique (Slice thickness = 5.0 mm, TR = 500 ms, TE = 12 ms, one excitation, flip angle = 120°, field of view = 42.0 × 42.0 mm^2^, matrix = 512 × 512, frequency = 123.194 Hz). During MRI scanning, participants rested quietly in the magnet bore in a supine position with their legs extended. All MRI data were transferred to a personal computer for analysis using specially designed image analysis software (sliceOmatic, Tomovision Inc., Montreal, Canada). Skeletal muscle tissue CSA data for the individual muscles located at 50% of thigh length were digitized. The MRI images of the right thighs were analyzed, because all participants selected the right legs to execute the single-leg stand-up test (described below). The following muscles were selected for segmentation: four heads of the quadriceps femoris (QF) (rectus femoris, RF; vastus lateralis, VL; vastus intermedius, VI; vastus medialis; VM), muscles composing the hamstrings (HAM) (biceps femoris short head, BFS; biceps femoris long head, BFL; semitendinosus, ST; semimembranosus, SM), adductors (sum of the adductor longus and adductor magnus, ADD), gracilis (GR), and sartorius (SA) (Fig 1). Visible fat at the muscle periphery (fat tissue within the muscle compartment), aponeurosis, blood vessels, nerves, and femur bone were excluded to the extent possible. In addition to the CSAs of individual muscles, the CSA of the whole thigh was also determined.

The CSA can be theoretically presented as a function of body mass to the two-third power [22]. Thigh muscle CSA has been reported to be strongly associated with body mass in middle-aged and elderly populations [23]. Accordingly, CSAs relative to the two-third power of body mass (CSA/BM^2/3^) were calculated to reduce the possible influence of body mass differences in the comparison of SG vs. USG. In addition, the percentage of the sum of individual muscle CSAs (i.e., total thigh muscle CSA) in the CSA of all tissues in the mid-thigh (i.e., whole thigh CSA) was also calculated.

### Stand-up test

Following the anthropometric measurements and MRI scanning, the stand-up test was conducted. The stand-up test was performed in accordance with the procedure described in a previous study [6, 24, 25]. Participants were seated on a 40-cm-height molded steel chair (depth, 40 cm; width, 60 cm: DANNO Manufacturing Co., Osaka, Japan) with their arms folded on the chest and feet spread to the width of the shoulders, with shins at an angle of 70 degrees to the floor. Participants were then asked to stand up from the sitting position, first on both legs and then on the right leg [6, 24, 25]. In the trails of the single leg stand-up test, the participants were asked to execute the task with the participants’ selected legs. All participants selected their right legs to execute the single-leg stand up task. Those who were able to stand up on a single leg without leaning back to gain momentum, and maintain a standing posture for 3 seconds, were allocated to SG, and those who could not were allocated to USG.

### Statistical analysis

Descriptive data are expressed as mean ± standard deviation (SD) with range (minimum-maximum). The Kolmogorov-Smirnov test was used to determine whether data followed a normal distribution pattern. Unpaired t test was used to test the significance of differences in measured variables between SG and USG. The Mann-Whitney U test was used if data were not distributed normally. Statistical significance was set at *P* < 0.05. As an index of the effect size, Cohen’s *d* values were reported with *P* values and interpreted as ‘trivial’ (< 0.20), ‘small’ (0.20–0.49), ‘medium’ (0.50–0.79), and ‘large’ (≥ 0.80) [26]. All statistical analyses were performed using SPSS Statistical Software, Version 25 (SPSS, Inc., Tokyo, Japan).

## Results

There were no significant differences in age and height between SG (n = 40) and USG (n = 27) (Table 1). Body mass, body mass index (BMI), and waist circumference were significantly greater in USG compared to SG. Only the CSA of ADD showed a significant difference between SG and USG (Table 2). However, CSA/BM^2/3^ values of QF, RF, VL, VI, VM, BFL, ADD, and total thigh muscle were significantly lower in USG compared to SG (Table 3). The percentage of total thigh muscle CSA in whole thigh CSA was significantly lower in USG (41.2 ± 7.0%, range: 25.4–55.4%) compared to SG (47.5 ± 5.3%, 37.6–59.1%) (*P* < 0.0001, *d* = 0.463).

**Table 1:** Physical characteristics of participants.

	SG, n = 40 mean ± SD (min—max)	USG, n = 27 mean ± SD (min—max)	*P* value	Effect size
Age, years	66.9 ± 5.5	68.7 ± 5.1	0.185	0.027
	(60 − 77)	(60 − 77)		
Height, cm	154.9 ± 4.8	156.8 ± 6.6	0.173	0.028
	(146.6 − 164.2)	(140.2 − 165.6)		
Body mass, kg	52.5 ± 7.3	59.6 ± 9.1	0.008	0.346
	(39.4 − 70.0)	(46.9 − 77.8)		
BMI, kg/m^2^	21.9 ± 2.9	24.2 ± 3.2	0.003	0.330
	(16.3 − 28.9)	(19.6 − 32.8)		
Waist circumference, cm	81.9 ± 8.0	91.0 ± 10.1	0.001	0.427
	(70.0 − 100.0)	(74.0 − 114.0)		

SG, successful group; USG, unsuccessful group

**Table 2:** Comparison of CSAs of individual mid-thigh muscles (cm^2^) between SG and USG.

	SG, n = 40 mean ± SD (min—max)	USG, n = 27 mean ± SD (min—max)	*P* value	Effect size
Total thigh muscle	75.9 ± 11.5	71.4 ± 10.6	0.110	0.039
	(52.7 − 100.8)	(51.1 − 95.3)		
QF	38.3 ± 6.3	35.7 ± 6.3	0.104	0.040
	(30.0 − 53.7)	(22.7 − 47.9)		
RF	4.4 ± 1.1	3.9 ± 0.9	0.057	0.054
	(2.9 − 8.0)	(0.9 − 5.6)		
VL	13.6 ± 3.0	12.7 ± 2.5	0.180	0.027
	(9.0 − 19.5)	(6.5 − 17.0)		
VI	12.0 ± 2.3	11.3 ± 2.5	0.205	0.025
	(8.0 − 17.9)	(7.6 − 16.7)		
VM	8.3 ± 1.4	7.9 ± 2.2	0.401	0.012
	(5.6 − 11.5)	(4.6 − 12.8)		
HAM	17.3 ± 3.1	17.5 ± 3.5	0.849	0.000
	(10.0 − 23.6)	(11.7 − 24.7)		
BFS	1.1 ± 0.4	1.1 ± 0.5	0.843	0.000
	(0.4 − 2.5)	(0.4 − 2.4)		
BFL	7.0 ± 1.4	6.7 ± 1.5	0.398	0.011
	(4.7 − 9.8)	(4.1 − 9.7)		
ST	4.2 ± 1.2	4.1 ± 1.2	0.740	0.002
	(2.1 − 7.7)	(2.0 − 6.8)		
SM	5.0 ± 1.5	5.5 ± 1.9	0.211	0.024
	(1.0 − 7.9)	(2.5 − 10.4)		
GR	2.1 ± 0.7	2.0 ± 0.6	0.588	0.004
	(0.8 − 3.7)	(1.2 − 3.5)		
SA	1.8 ± 0.5	1.8 ± 0.6	0.611	0.005
	(0.8 − 3.2)	(1.0 − 3.7)		
ADD	16.4 ± 4.3	14.3 ± 3.2	0.039	0.246
	(8.6 − 25.8)	(8.1 − 22.5)		

SG, successful group; USG, unsuccessful group; QF, quadriceps femoris; RF, rectus femoris; VL, vastus lateralis; VI, vastus intermedius; VM, vastus medialis; HAM, hamstrings; BFS, biceps femoris short head; BFL, biceps femoris long head; ST, semitendinosus, SM, semimembranosus, GR, gracilis; SA, sartorius; ADD, adductors

**Table 3:** Comparison of CSA/BM^2/3^ values of individual mid-thigh muscles (cm^2^/kg^2/3^) between SG and USG.

	SG, n = 40 mean ± SD (min—max)	USG, n = 27 mean ± SD (min—max)	*P* value	Effect size
Total thigh muscle	5.416 ± 0.584	4.689 ± 0.496	< 0.001	0.528
	(4.414 − 6.534)	(3.725 − 5.521)		
QF	2.734 ± 0.350	2.348 ± 0.356	< 0.001	0.455
	(2.128 − 3.550)	(1.655 − 3.001)		
RF	0.315 ± 0.073	0.257 ± 0.060	0.001	0.407
	(0.219 − 0.517)	(0.060 − 0.399)		
VL	0.967 ± 0.170	0.832 ± 0.149	0.001	0.357
	(0.666 − 1.404)	(0.478 − 1.170)		
VI	0.860 ± 0.151	0.740 ± 0.152	0.002	0.367
	(0.611 − 1.186)	(0.530 − 1.108)		
VM	0.592 ± 0.088	0.519 ± 0.136	0.009	0.299
	(0.431 − 0.791)	(0.324 − 0.796)		
HAM	1.240 ± 0.202	1.147 ± 0.189	0.064	0.052
	(0.865 − 1.701)	(0.788 − 1.466)		
BFS	0.079 ± 0.028	0.073 ± 0.030	0.446	0.009
	(0.031 − 0.171)	(0.021 − 0.165)		
BFL	0.503 ± 0.095	0.440 ± 0.076	0.006	0.314
	(0.359 − 0.772)	(0.296 − 0.567)		
ST	0.300 ± 0.079	0.270 ± 0.075	0.130	0.035
	(0.161 − 0.476)	(0.138 − 0.418)		
SM	0.358 ± 0.104	0.364 ± 0.118	0.839	0.001
	(0.066 − 0.535)	(0.184 − 0.636)		
GR	0.151 ± 0.043	0.133 ± 0.029	0.070	0.050
	(0.060 − 0.235)	(0.085 − 0.208)		
SA	0.125 ± 0.031	0.120 ± 0.031	0.498	0.007
	(0.065 − 0.217)	(0.072 − 0.206)		
ADD	1.166 ± 0.270	0.941 ± 0.177	0.003	0.414
	(0.687 − 1.740)	(0.561 − 1.234)		

SG, successful group; USG, unsuccessful group; QF, quadriceps femoris; RF, rectus femoris; VL, vastus lateralis; VI, vastus intermedius; VM, vastus medialis; HAM, hamstrings; BFS, biceps femoris short head; BFL, biceps femoris long head; ST, semitendinosus, SM, semimembranosus, GR, gracilis; SA, sartorius; ADD, adductors

## Discussion

The present study is the first to investigate differences in individual thigh muscle CSAs between older women who can stand up from a 40-cm-height chair on a single leg and those who cannot. Our major findings were that 1) ADD was the only muscle group with a significant difference in CSA between SG and USG, and 2) the CSA/BM^2/3^ values of BFL, ADD, and four heads of QF (RF, VL, VI, and VM) were significantly lower in USG compared to SG. These results partially support our hypothesis and suggest that the reduced CSA/BM^2/3^ values of ADD and BFL as well as QF are linked to the augmentation of the difficulty of standing up from a 40-cm-height chair on a single leg in older women.

The observed difference in the CSA of ADD between SG and USG may be due to the contribution of this muscle group to the movements that involve hip extension during the stand-up task. Previous findings based on T2 [17], the signal intensity of MRI images [16], and EMG activities [18] indicate increased activation of the adductors in the execution of movements involving hip extension. In addition, Montgomery et al. [27] reported that, based on an electromyographic analysis of hip and knee musculature during running, the adductor magnus afforded pelvic stabilization while assisting with hip flexion and extension. Furthermore, Kubo et al. [28] examined the effect of squat training with different depths on lower limb muscle volumes and observed a greater hypertrophic change in the adductors in the full-squat condition than in the half-squat condition. These reports suggest that the adductors may be activated more in a task that requires greater hip extension. From the anatomical viewpoint, the adductor magnus is activated during extension of the thigh at the hip joint [29]. In the present study, the CSA of ADD was determined as the sum of CSAs of the adductor magnus and adductor longus. Thus, we cannot determine whether the observed difference in the CSA of ADD between the two groups is attributed to the adductor magnus alone. However, Ito et al. [30] reported that the CSA of the muscle belly and the weight of the adductor magnus were the second largest among individual lower limb muscles in an adult cadaver (male, 58-years-old). Thus, in USG, the CSA of ADD appears to mainly reflect the CSA of the adductor magnus, and hence, a smaller CSA of ADD may be the reason for failing to execute the stand-up task.

The CSA/BM^2/3^ values of the four heads of QF and BFL, as well as ADD, were significantly greater in SG than in USG. The CSA and force generation capability of QF have been reported to be associated with repeated stand-up performance in older adults [21]. The present study provided additional evidence that the reduced CSA/BM^2/3^ of each of the four heads of QF is associated with a greater difficulty of executing the single-leg stand-up task. Interestingly, however, among the muscles composing HAM, only BFL showed a significant difference in CSA/BM^2/3^ between SG and USG. To our knowledge, few studies have addressed how each of the four muscles composing HAM is associated with stand-up performance. According to Bourne et al. [31], hip extension training as compared to Nordic hamstring exercise produced a greater hypertrophic change in the biceps femoris long head. They reported that mean values of percentage changes in the volume of the biceps femoris short head and semitendinosus were higher after Nordic hamstring exercise compared to hip extension exercise, although the differences were not significant. Furthermore, an electromyographic study reported that, while the biceps femoris short head plays a role in knee flexion alone, the other members of the hamstrings are involved in hip extension [27]. Similarly, Ono et al. [32] reported that EMG activities of the biceps femoris long head, as well as semimembranosus muscles, during hip extension were significantly higher compared to those of semitendinosus muscles. Based on these findings, the biceps femoris long head appears to mainly contribute to the execution of tasks including hip extension. Thus, the low CSA/BM^2/3^ of this muscle might explain the greater difficulty of executing the single-leg stand-up task.

Even if the assumption described above is accurate, it is unclear why CSA/BM^2/3^ values of SM and ST did not show a significant difference between USG and SG. Based on the aforementioned findings derived from EMG activities [27, 32], SM and ST are also activated during hip extension. Thus, the CSA/BM^2/3^ values of the two muscle groups were expected to be significantly smaller in USG compared to SG. The reason for the lack of differences between the two groups in the CSA/BM^2/3^ values of SM and ST is unknown, but might involve the influence of the thigh slice level used in MRI measurements. In the present study, MRI images obtained at the mid-thigh were used to determine the CSAs of individual thigh muscles. The anatomical CSA of BFL becomes almost the maximum around the mid-thigh, but the corresponding values for SM and ST appear at distal and proximal levels, respectively, of the femur length [33]. The anatomical CSAs of SM and ST at the mid-thigh are smaller than that of the BFL [33], as observed here (Table 2). Among the four muscles composing HAM, however, SM has the largest muscle volume [33, 34]. Thus, it is possible that at least for SM, the CSA determination at the mid-thigh did not reflect adequately the size of this muscle and so it might not have significant differences between the two groups.

The percentage of total thigh muscle CSA in whole thigh CSA was lower in USG compared to SG. In addition, the mean values of body mass, BMI, and waist circumference were significantly greater in USG compared to SG. Given that no significant differences were observed in the CSAs of all analyzed muscle groups except for ADD, the thigh composition of participants in USG may be characterized by similar muscle mass and greater fat mass as compared to those in SG. In older adults, body fat mass is inversely associated with lower extremity performance, such as repeated chair-stand and 6-m walk [35–39]. Bouchard et al. [35] examined the associations of sarcopenia/obesity with physical capacity in older men and women and concluded that obesity per se contributed more to lower global physical capacity compared to sarcopenia. In addition, Visser et al. [39] reported that in men and women aged 70–79 years, the most important body composition component related to lower extremity performance was thigh muscle CSA in men, but total body fat mass in women. Furthermore, it is known that obesity is related to postural instability [23, 40, 41]. According to Ochi et al. [23], both abdominal visceral fat area and QF CSA-corrected body weight are associated with static postural instability. In older adults, balance is a significant independent predictor for sit-to-stand performance [42]. Thus, in addition to lower CSA/BM^2/3^ values of muscle groups contributing to the execution of the single-leg stand-up task, differences in body composition between SG and USG may be also associated with the success/failure to complete the task.

The limitations of the present study include the method used for muscle CSA measurement. Muscle CSA was determined using a single axial MRI image at the mid-thigh. A previous study using cadavers reported that thigh CSA was maximal at the midpoint of the thigh length [43]. As described earlier, however, this does not mean that the distribution of CSA for each of the individual muscles along the thigh length peaks at the midpoint of the thigh length. The slice level of the thigh, at which maximal CSA is obtained, varies considerably among individual muscles (between 20% and 80% of the femur length) [33]. In addition, muscle volume rather than anatomical muscle CSA is more appropriate for evaluating muscle strength [44, 45]. According to Hogrel et al. [33], a single slice CSA which provided the best estimate of QF muscle volume was obtained at 50% of femur length, but the corresponding slice levels for HAM and ADD were 40% and 60% of femur length, respectively. Thus, we cannot rule out the possibility that, when muscle volume is used as a parameter representing muscle size, muscles associating with the success/failure of the stand-up task may differ from those observed in the present study. Furthermore, we have no information concerning the physiological reasons yielding the group differences observed in the CSAs of ADD and BFL, as well as the four heads of QF. In addition, whether the findings obtained in this study can be applied to older men and/or other generations remains question. In order to generalize our findings, further study will be needed to clarify these aspects.

In conclusion, the present study showed that the smaller CSA/BM^2/3^ values of ADD and BFL as well as QF are associated with a greater difficulty of standing up from a 40-cm-height chair on a single leg in older women. This may be explained in terms of the anatomical function of the adductors and biceps femoris long head, which contribute to hip extension movement involved in transitioning from a sitting position to a standing position during the stand-up task. The findings obtained here also suggest the merits of performing multi-joint exercises, such as leg press and hip/knee extension exercises, rather than single-joint knee extension exercise, for improving the ability of older women to stand up from a chair without support.

## Supporting information

S1 File (XLSX)
